# High-dose intravenous iron significantly reduces the risk of red blood cell transfusion and improves postoperative hemoglobin levels after cardiac surgery: A systematic review of randomized controlled trials

**DOI:** 10.1371/journal.pone.0336773

**Published:** 2025-11-13

**Authors:** Lei Wang, Chang Han Ma, Si Yuan Yang, Zheng Gang Zhang

**Affiliations:** Department of Cardiac Surgery, The Affiliated Hospital of Guizhou Medical University, Guiyang, Guizhou Province, China; Ataturk University Faculty of Medicine, TÜRKIYE

## Abstract

**Background:**

High-dose intravenous iron supplementation offers substantial hematologic protective benefits in clinical practice; however, its efficacy in enhancing blood protection during cardiac surgery remains uncertain. The present study aimed to investigate the effects and safety of high-dose intravenous iron as an optimal blood management strategy for patients undergoing cardiac procedures.

**Methods:**

Major databases, including PubMed, Embase, and Cochrane, were searched on June 20, 2025, for randomized controlled trials (RCTs) comparing red blood cell transfusion rates in adult patients undergoing high-dose intravenous iron supplementation versus those receiving control therapy (placebo) following cardiac surgery. The secondary outcome measures included postoperative hemoglobin levels, length of hospital stay, and incidence of adverse events.

**Results:**

Seven RCTs involving 975 subjects were identified in the database search. Compared with the control group (placebo), high-dose intravenous iron significantly decreased the rate of postoperative red blood cell transfusion among patients undergoing cardiac surgery (risk ratio 0.69, 95% confidence interval [CI] 0.52–0.91, P = 0.009, I^2^ = 61%, n = 975, certainty of evidence: moderate). Furthermore, one week or more following surgery, administration of high-dose intravenous iron resulted in a significant increase in postoperative hemoglobin levels (mean difference 0.71, 95% CI 0.41 to 1.01, P < 0.00001, n = 907, I^2^ = 63%, certainty of evidence: moderate). Significant differences between the groups were not observed for the other outcome measures, including mortality, infection rates, and cardiovascular events.

**Conclusions:**

High-dose intravenous iron supplementation during the perioperative period of cardiac surgery significantly reduces the risk of red blood cell transfusion and enhances postoperative hemoglobin levels. Although the present study demonstrated a favorable safety profile for intravenous iron administration, the limitations of the present meta-analysis necessitate continued vigilance regarding potential drug-related risks associated with intravenous iron therapy. Systematic review protocol: CRD420251069827 (PROSPERO).

## Introduction

Perioperative anemia is a prevalent issue among patients undergoing cardiac surgery. Perioperative anemia can be caused by various factors as follows: hemodilution and blood cell destruction associated with cardiopulmonary bypass; blood loss due to surgical trauma and coagulation dysfunction; and suppression of erythropoiesis resulting from postoperative inflammation [1,2]. Previous studies have indicated that the incidence of postoperative anemia in patients undergoing cardiac surgery ranges from 29% to 94% [1]. Anemia is directly correlated with the prognosis of patients following cardiac surgery and is associated with increased mortality, prolonged hospital stays, and higher treatment costs [3]. Traditionally, anemia has been primarily managed through transfusion; however, evidence suggests that, in addition to the risks associated with transfusion, the administration of even a single unit of red blood cells may increase the long-term mortality rates of patients [4]. There has been a sustained clinical effort to minimize the use of blood products and enhance patient outcomes through effective blood management protocols [5]. The use of iron supplements for blood protection during the perioperative period in cardiac surgery constitutes a critical component of these protocols and has garnered significant attention over the years [6].

Oral iron supplements are associated with gastrointestinal adverse effects and an increased risk of enteritis [7,8]. In contrast, intravenous iron supplementation circumvents gastrointestinal absorption, thereby preventing common gastrointestinal side effects and facilitating rapid replenishment of iron levels and enhancement of hemoglobin concentrations [9]. Patients undergoing cardiac surgery often lack sufficient time to take oral iron supplements to rectify iron deficiency and anemia; in addition, many individuals exhibit suboptimal responses to oral iron therapy due to comorbidities [10]. Consequently, intravenous iron is more appropriate for managing anemia related to the perioperative period in cardiac surgery patients. During emergency procedures, high-dose iron supplements (1000–1500 mg) can be rapidly administered to restore iron levels and improve patient prognosis [11]. A previous study has demonstrated that high-dose intravenous ferric carboxymaltose (1000 mg for patients weighing over 50 kg) is more effective than low-dose sucrose iron (up to 600 mg) in rapidly correcting iron deficiency anemia in gynecological patients with preoperative anemia [12]. Another study has reported that high-dose intravenous iron (500–1000 mg) is effective and safe for blood management in orthopedic surgery patients, reducing the need for transfusions and mitigating decreased hemoglobin levels [13]. Thus, high-dose intravenous iron provides significant protective advantages in clinical blood management; however, its efficacy as a blood-protective agent in cardiac surgery requires further investigation.

Numerous systematic reviews have demonstrated that intravenous iron supplementation is effective for blood protection during the perioperative period in cardiac surgery. However, the studies included in these reviews do not distinguish between different doses of intravenous iron preparations, preventing adequate assessment of the efficacy and safety of high-dose intravenous iron supplementation. Furthermore, some meta-analyses have included observational studies and studies involving combined adjuvant therapies, such as iron supplementation in conjunction with erythropoietin and folic acid, which may introduce bias and heterogeneity [14–17].

Therefore, the present meta-analysis examined the efficacy and safety of high-dose intravenous iron supplementation, without accompanying adjuvant therapy, in the context of perioperative blood management for cardiac surgery. The aim of the present study was to provide evidence-based recommendations for the optimal use of intravenous iron agents in patients undergoing cardiac surgery.

## Methods

### Ethics statement

Although this study involved human participants, it was a systematic review and only involved secondary data analysis. Thus, approval from an ethics review committee was not needed.

### Search strategy

The present meta-analysis followed the Preferred Reporting Items for Systematic Reviews (PRISMA) guidelines [18]. The protocol was registered with the International Prospective Systematic Review Registry (PROSPERO, registration number: CRD420251069827, registrant: Lei Wang, link: http://dx.doi.org/10.17504/protocols.io.x54v95lkz3e/v1). A comprehensive literature search across the PubMed, Embase, and Cochrane databases was conducted to identify randomized controlled trials (RCTs) comparing intravenous iron supplementation with a placebo control following cardiac surgery. The search strategy incorporated a combination of medical subject headings (MeSH) pertinent to iron preparations and cardiac surgery, along with relevant free-text keywords. The following keywords were used with Boolean operators in the literature search: “iron” OR “ferric carboxymaltose” OR “iron isomaltoside 1000” OR “saccharated ferric oxide” OR “ferric gluconate” OR “iron-dextran complex” AND “thoracic surgery” OR “coronary artery bypass” OR “heart valve prosthesis implantation”. The search was limited to peer-reviewed articles from the establishment of each database to June 20, 2025. No restrictions were applied regarding publication date, language, or sample size. For studies published in languages other than English that initially appeared eligible based on their English titles and abstracts, Google Translate was utilized to assess the full text. The search details for the databases are presented in Supplementary S1 Table.

### Selection of included studies

After deduplication of the retrieved literature using Endnote (21.5 version; London, Clarivate Analytics), two reviewers (Lei Wang and Chang Han Ma) independently screened the titles and abstracts of the resulting studies to identify potentially eligible studies. The reviewers independently conducted full-text reviews to determine whether the studies met the established inclusion criteria. In cases of disagreements between the reviewers, discussions or consultations with a third reviewer (Zheng Gang Zhang) were held to reach a consensus. The following inclusion criteria were defined on the basis of the Patients, Interventions, Comparators, Outcomes, and Study design (PICOS) framework: (1) adult patients aged 18 years or older, with or without anemia, undergoing any type of cardiac surgery, including coronary artery bypass grafting, valve replacement surgery, or a combination of both; (2) high-dose intravenous iron supplementation in the perioperative period, irrespective of the type and duration of iron preparations; (3) studies utilizing a placebo as a control; (4) primary outcome measure being the proportion of patients requiring postoperative red blood cell transfusion; and (5) peer-reviewed RCTs. With regard to iron supplementation, a total intravenous iron dose of 500 mg or lower was categorized as low dose, and a total intravenous dose greater than 500 mg was classified as high dose. The 500 mg cutoff was determined on the basis of a recommendation of at least 500 mg for newly developed intravenous iron compounds. The high-dose intravenous iron supplementations included FCM (500 mg and 1000 mg), ferric derisomaltose (FDI) (1000 mg), and isomaltose (IS) (600 mg) [19–21]. In addition to the primary outcome, other relevant outcomes were assessed, including the number of transfused red blood cell units.

The exclusion criteria were as follows: (1) case reports, conference abstracts, non-randomized studies, observational studies, or review articles; (2) low-dose iron preparations; (3) studies involving minors; (4) patients undergoing transcatheter aortic valve implantation; (5) studies employing oral iron preparations or combined with other adjunct therapies (such as erythropoietin, vitamins, or folic acid); and (6) studies lacking the specified outcomes or insufficient statistical data. For repetitively published studies or studies that used the same patient population, the most comprehensive or recently published study was included in the meta-analysis, while the others were not included.

**Fig 1:**
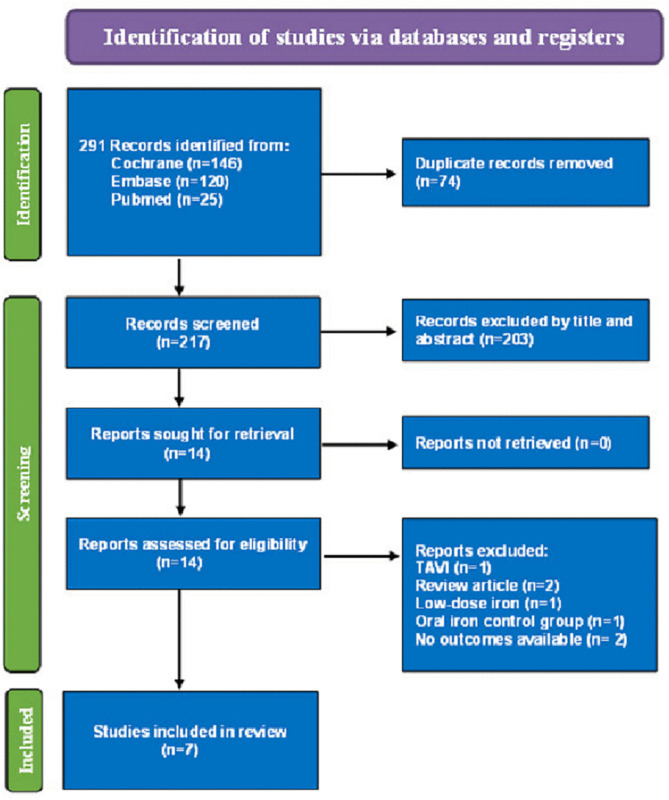
Preferred Reporting Items for Systematic Reviews and Meta-Analyses (PRISMA) flow diagram for the identification and selection of studies. TAVI, transcatheter aortic valve implantation.

A preliminary literature search identified a total of 291 studies from three databases. The Preferred Reporting Items for Systematic Reviews and Meta-Analyses (PRISMA) flowchart shown in Fig 1 illustrates the study selection process, which ultimately included seven studies for systematic evaluation [22–28]. The seven RCTs involved a total of 975 subjects (Table 1). Among the included RCTs, three included patients without preoperative anemia [24–26], three included patients with preoperative anemia [23,27,28], and one included a mixed patient population with preoperative anemia and no anemia [22]. The average age of the subjects ranged from 53.2 to 72.2 years, with the male-to-female ratio varying from 0.6 to 9.2 across the studies. Five studies reported body mass index (BMI) values, with averages between 22.80 and 29.70 kg/m^2^ [22–26]. Six studies documented preoperative hemoglobin levels, averaging between 10.03 and 14.65 g/dL [23–28]. All studies utilized high doses of intravenous iron, including 1000 mg in five studies [23–27], greater than 1000 mg in one study [22], and a minimum of 621 mg in one study [28]. The control group for all studies received a placebo (saline).

**Table 1:** General Characteristics of the Included Studies.

Study (year)	Patients	Sample Size (n)*	Mean age (y)*	Males: Females (n)*	Mean BMI^a^ (kg/m^2^)*	Mean Hb^c^ (g/dL)*	Dose of iron (mg)	Control
2022Song	Anemia/Non-anemia	103/101	61.00/63.00	39:64/42:59	23.80/23.50	NR	≧1000	Placebo
2023Kim	Anemia	43/43	70.60/72.20	31:12/30:13	23.50/22.80	11.30/11.10	1000	Placebo
2023Houry	Non-anemia	97/98	64.30/66.35	73:24/72:26	27.05/26.63	14.10/13.90	1000	Placebo
2023Friedman	Non-anemia	102/98	62.50/62.70	92:10/83:15	28.90/29.70	14.65/14.34	1000	Placebo
2015Johansson	Non-anemia	30/30	65.00/65.00	26:4/26:4	28.00/28.00	14.25/13.98	1000	Placebo
2022Shokri	Anemia	40/40	58.35/60.08	18:22/25:15	NR^b^	12.76/10.03	1000	Placebo
2019Xu	Anemia	75/75	55.50/53.20	31:44/36:39	NR	14.13/13.86	≧621	Placebo

^a^BMI, body mass index; ^b^NR, not reported; ^c^Hb, hemoglobin; *Presented as intervention vs control groups.

### Quality assessment of the included studies

Two reviewers independently used the Cochrane Risk of Bias 2 (RoB 2) tool to assess the methodological quality and risk of bias of the included studies [29]. Any disagreements were resolved through discussion or consultation with a third reviewer. The Cochrane RoB 2 tool evaluates five domains—randomization process, deviations from the intended intervention, missing outcome data, outcome measurement, and selection of reported results. Each domain is assessed for its risk of bias, categorized as low risk, some concerns, or high risk. Based on the assessment results of each domain, an overall risk of bias judgment was determined for each study.

**Fig 2:**
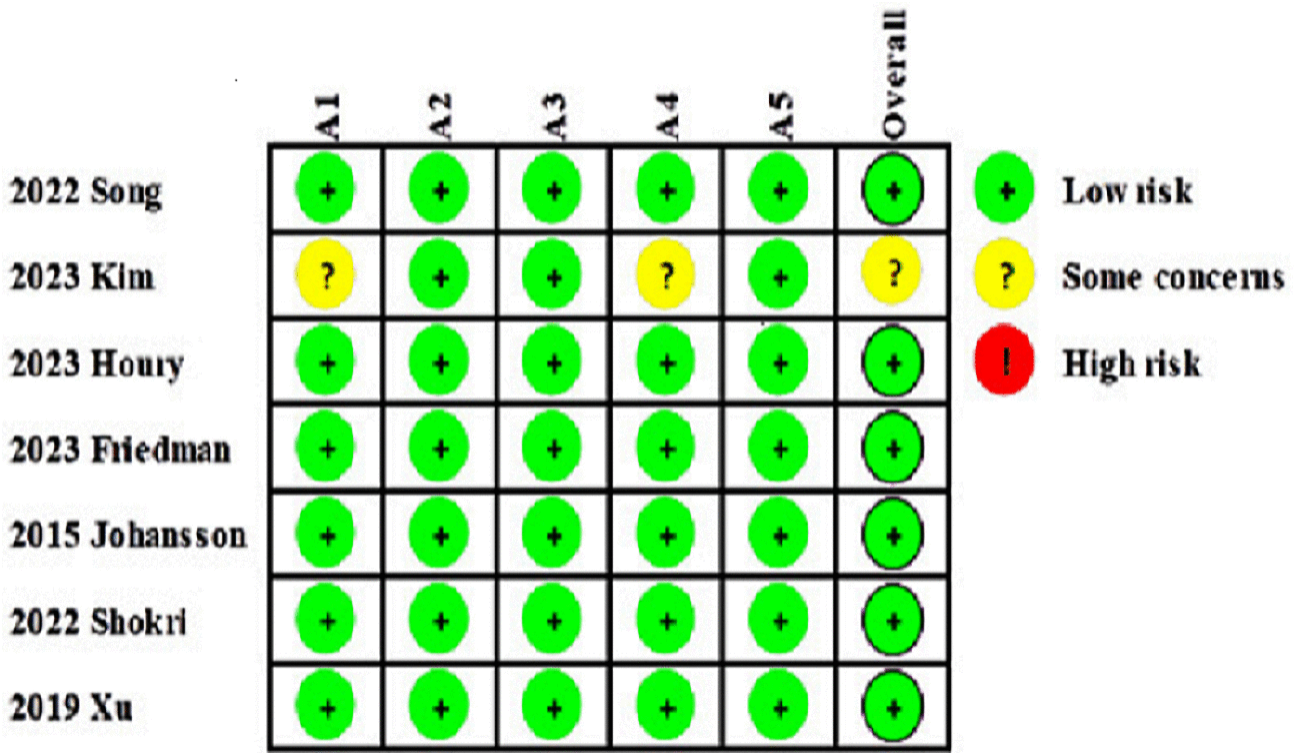
Risk of bias for each study. A1, Randomization process; A2, Deviations from intended interventions; A3, Missing outcome data; A4, Measurement of the outcome; A5, Selection of the reported result.

Among the seven included RCTs, six were evaluated based on the rate of red blood cell transfusion [22–26,28], and one study, which did not report this rate, was assessed based on postoperative hemoglobin levels [27]. With the exception of one study, for which the reviewers had concerns regarding the overall risk of bias, the remaining studies were determined to be at low risk of bias (Fig 2).

### Data extraction

Two reviewers independently collected available data from the included studies, addressing any discrepancies through discussion or consultation with a third reviewer. The characteristics of the included studies were extracted, including the first author’s name, publication year, and the characteristics of the subjects (such as average age, sex, BMI, preoperative hemoglobin levels, and intervention details). In terms of outcome measurements, data on red blood cell transfusion rates, postoperative hemoglobin levels, length of hospital stay, and adverse events (including death, infections, and cardiovascular events) were extracted. The red blood cell transfusion rate was defined as the proportion of patients receiving transfusions in both the intervention and placebo groups following the administration of intravenous iron agents and surgical treatment. Postoperative hemoglobin values were collected for periods within one week after surgery and beyond; if multiple outcome measurements occurred within the specified timeframe, the earliest or available data after surgery were utilized. Cardiovascular events were defined as incidents of stroke, myocardial infarction, and atrial fibrillation, while infections were defined as any form of infection occurring after surgery, including pulmonary infections and those related to surgical incisions. In cases where data was missing, the authors of the relevant article were contacted; if the authors were unavailable, alternative methods to obtain the data were explored. However if data could not be obtained, the data was recorded as missing.

### Data analysis

The primary outcome measure of the present meta-analysis was the risk ratio (RR) of transfusion rates between the high-dose intravenous iron group and the control group following cardiac surgery. Secondary outcome measures included the mean difference (MD) in post-operative hemoglobin levels, the MD in the length of hospital stay, and the RR of adverse events (including death, cardiovascular events, and infections). The present meta-analysis used 95% CI to present the MD for continuous variables and the RR for binary variables. The I^2^ statistic was utilized to evaluate study heterogeneity, with values exceeding 75% indicating significant heterogeneity. Regardless of the degree of heterogeneity, a random-effects model (DerSimonian and- Laird method) was applied to synthesize effect sizes. For the primary outcome measure of red blood cell transfusion rates, the studies were categorized based on preoperative hemoglobin status to distinguish between groups with and without anemia. Subgroup analyses were performed to identify potential sources of heterogeneity. For outcome indicators exhibiting significant heterogeneity, subgroup analyses based on preoperative anemia status, type of iron preparation, and timing of administration were performed to elucidate the potential sources of this heterogeneity. Sensitivity analyses were performed for all outcome measures by sequentially excluding each study to assess the robustness of the findings. In studies involving five or more participants, funnel plots were utilized for the visual assessment of publication bias. The results, combined effect estimates, and overall composite effects of each study are illustrated by forest plots, which were constructed using RevMan (version 5.4; Copenhagen, The Cochrane Collaboration). P < 0.05 was considered to indicate statistical significance.

### Certainty of evidence

The quality of evidence for all outcomes was assessed using the Grading of Recommendations Assessment, Development, and Evaluation (GRADE) approach [30], which involves five domains, namely, risk of bias, inconsistency, indirectness, imprecision, and publication bias. Using this approach, the quality of outcome evidence was categorized as very low, low, moderate, or high. In addition, an importance rating was assigned to each assessed outcome.

## Results

### Primary outcome: red-cell transfusion rate

**Fig 3:**
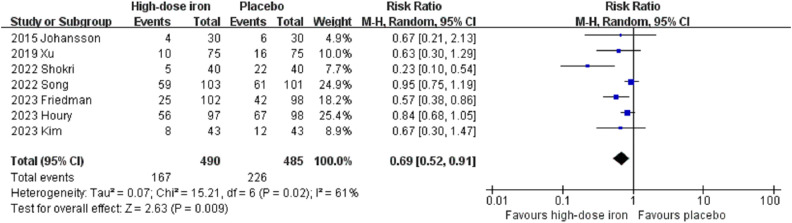
Forest plots of the included studies showing the overall blood transfusion rate between the high-dose intravenous iron group and the placebo group. CI, confidence interval; M-H, Mantel-Haenszel.

Six studies involving a total of 490 patients in the high-dose intravenous iron group and 485 patients in the placebo group reported the rate of red blood cell transfusion. The meta-analysis revealed that the risk of red blood cell transfusion was significantly lower in the high-dose intravenous iron group compared with the placebo group (RR 0.69, 95% CI 0.52 to 0.91, P = 0.009, I^2^ = 61%) (Fig 3). Sensitivity analysis, which involved sequentially excluding each study, yielded consistent results, indicating that no single study exerted a disproportionately large influence on the overall findings (S2 Table in S1 File). The funnel plot exhibited slightly diminished symmetry, suggesting an increased likelihood of publication bias (S1 Fig). Subgroup analysis based on preoperative anemia status demonstrated that high-dose intravenous iron significantly reduced the risk of red blood cell transfusion in patients with preoperative anemia (RR 0.47, 95% CI 0.24 to 0.90, P = 0.02, I^2^ = 51%, 3 RCTs, 316 participants) and in those without preoperative anemia (RR 0.73, 95% CI 0.54 to 0.99, P = 0.04, I^2^ = 34%, 3 RCTs, 455 participants) (S2 Fig). Subgroup analysis revealed no significant differences among the subgroups (P = 0.23).

### Secondary outcome measure: postoperative hemoglobin value

**Fig 4:**
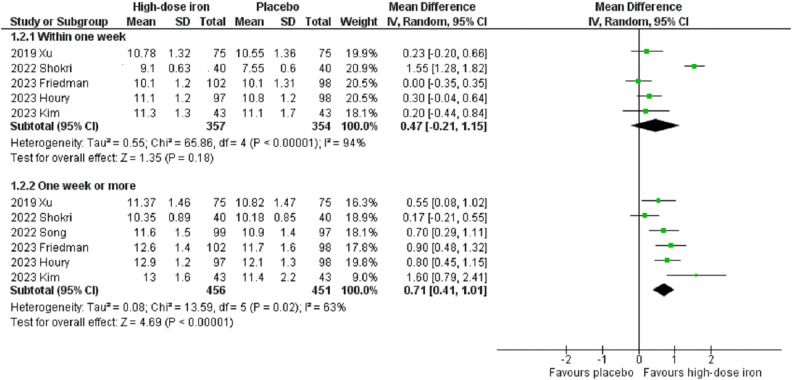
Forest plots of included studies showing the mean difference in postoperative hemoglobin values between the high-dose intravenous iron group and the placebo group. CI, confidence interval; M-H, Mantel-Haenszel.

The comparison of postoperative hemoglobin levels between the high-dose intravenous iron group and the placebo group was performed at two time points—within one week after surgery and one week or more after surgery (Fig 4). Although no significant differences in hemoglobin levels were observed between the groups within the first week after surgery, the hemoglobin levels in the high-dose intravenous iron group were marginally greater than those in the placebo group (MD 0.47, 95% CI −0.21 to 1.15, P = 0.18, I^2^ = 94%, 5 RCTs, 711 participants). In contrast, at one week or more after surgery, the hemoglobin levels in the high-dose intravenous iron group were significantly greater than those in the placebo group (MD 0.71, 95% CI 0.41 to 1.01, P < 0.00001, I^2^ = 63%, 6 RCTs, 907 participants). For the outcome indicator with significant heterogeneity (hemoglobin value within one week after surgery), subgroup analysis based on preoperative anemia status demonstrated that high-dose intravenous iron significantly increased postoperative hemoglobin values in patients without anemia (MD 0.84, 95% CI 0.57 to 1.11, P < 0.00001, I^2^ = 0%, 2 RCTs, 395 participants), while there was no significant change in postoperative hemoglobin values in patients with anemia (MD 0.68, 95% CI −0.33 to 1.69, P = 0.19, I^2^ = 94%, 3 RCTs, 316 participants) (S3 Fig). Subgroup analysis based on the timing of administration indicated that patients receiving treatment preoperatively had no significant change in postoperative hemoglobin values (MD 0.60, 95% CI −0.54 to 1.74, P = 0.30, I^2^ = 96%, 3 RCTs, 366 participants), whereas significant changes in postoperative hemoglobin values were observed in patients who received postoperative treatment (MD 0.27, 95% CI 0.01 to 0.54, P = 0.04, I^2^ = 0%, 2 RCTs, 345 participants) (S4 Fig). Subgroup analyses revealed no significant differences between groups based on anemia status (P = 0.77) or timing of administration (P = 0.59). The results of the sensitivity analysis on postoperative hemoglobin levels were consistent (S3 and S4 Tables in S1 File). Furthermore, the funnel plot indicated an asymmetric distribution, suggesting a potential presence of publication bias (S5 Fig).

### Secondary outcome measures: length of hospital stay and adverse events (mortality, infection, and cardiovascular events)

**Fig 5:**
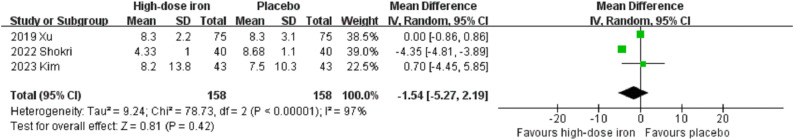
Forest plots of included studies showing the mean difference in length of hospital stay between the high-dose intravenous iron group and the placebo group. CI, confidence interval; M-H, Mantel-Haenszel.

**Fig 6:**
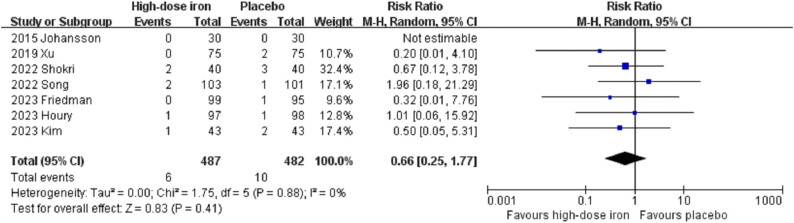
Forest plots of included studies showing the death rate between the high-dose intravenous iron group and the placebo group. CI, confidence interval; M-H, Mantel-Haenszel.

**Fig 7:**
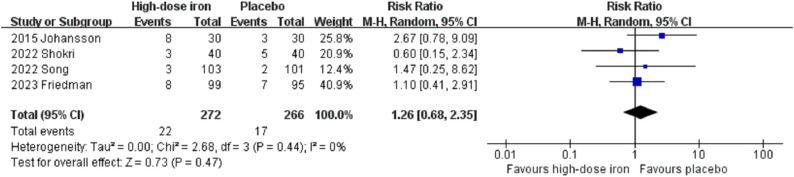
Forest plots of included studies showing the infection rate between the high-dose intravenous iron group and the placebo group. CI, confidence interval; M-H, Mantel-Haenszel.

**Fig 8:**
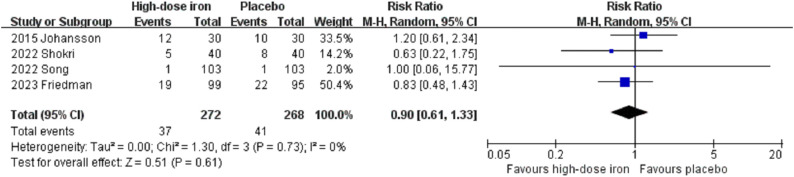
Forest plots of included studies showing the rate of cardiac events between the high-dose intravenous iron group and the placebo group. CI, confidence interval; M-H, Mantel-Haenszel.

The present meta-analysis revealed no significant differences between the two groups regarding the length of hospital stay (MD −1.54, 95% CI −5.27 to 2.19, P = 0.42, I^2^ = 97%, 3 RCTs, 316 participants) (Fig 5). Moreover, no significant differences were observed in mortality (RR 0.66, 95% CI 0.25 to 1.77, P = 0.41, I^2^ = 0%, 7 RCTs, 969 participants) (Fig 6), infection rate (RR 1.26, 95% CI 0.68 to 2.35, P = 0.47, I^2^ = 0%, 4 RCTs, 538 participants) (Fig 7), or incidence of cardiovascular events (RR 0.90, 95% CI 0.61 to 1.33, P = 0.61, I^2^ = 0%, 4 RCTs, 540 participants) (Fig 8). For the outcome indicator with significant heterogeneity, namely, length of hospital stay, the subgroup analysis based on the timing of administration (preoperative or postoperative) and the type of intravenous iron preparation (FCM or IS) demonstrated that there was no significant change in the length of hospital stay among patients receiving preoperative FCM (MD −2.50, 95% CI −7.27 to 2.27, P = 0.30, I^2^ = 73%, 2 RCTs, 166 participants) or postoperative IS (95% CI −0.86 to 0.86, P = 1.00, 1 RCT, 150 participants). Subgroup analysis revealed no significant differences among the subgroups (P = 0.31) (S6 Fig). Additionally, the sensitivity analyses for length of hospital stay and adverse events yielded consistent results (S5-S8 Tables in S1 File). The funnel plot for mortality indicated a symmetric distribution, indicating a low risk of publication bias (S7 Fig).

### Certainty of evidence

The quality of evidence was assessed for all outcomes. Most outcomes were classified as moderate quality, while a smaller proportion was categorized as either low or high quality (Table 2).

**Table 2:** Summary of outcomes and certainty of evidence based on the Grading of Recommendations Assessment, Development and Evaluation (GRADE) approach.

Outcomes	Certainty assessment					Sensitivity analysis	Relative effect	№ of participants	Certainty of the evidence	Importance
	A1^a^	A2^b^	A3^c^	A4^d^	A5^e^		(95% CI^f^)	(studies)	(GRADE)	
Red blood cell transfusion rate	**+**	**+**	**+**	**+**	**－**	Consistent	RR^g^ 0.69	975	⨁⨁⨁◯	Critical
							(0.52 to 0.91)	(7 RCTs)	Moderate	
Postoperative hemoglobin level (within one week)	**+**	**+**	**+**	**－**	**－**	Consistent	MD^h^ 0.47	711	⨁⨁◯◯	Not important
							(-0.21 to 1.15)	(5 RCTs)	Low	
Postoperative hemoglobin level (one week or more)	**+**	**+**	**+**	**+**	**－**	Consistent	MD 0.71	907	⨁⨁⨁◯	Important
							(0.41 to 1.01)	(5 RCTs)	Moderate	
Length of hospital stay	+	+	+	－	Unable to assess	Consistent	MD -1.54	316	⨁⨁◯◯	Not important
							(-5.27 to 2.19)	(3 RCTs)	Low	
Death rate	+	+	+	+	+	Consistent	RR 0.66	969	⨁⨁⨁⨁	Critical
							(0.25 to 1.77)	(7 RCTs)	High	
Infection rate	+	+	+	－	Unable to assess	Consistent	RR 1.26	538	⨁⨁⨁◯	Important
							(0.68 to 2.35)	(4 RCTs)	Moderate	
Cardiac events rate	+	+	+	－	Unable to assess	Consistent	RR 0.90	540	⨁⨁⨁◯	Important
							(0.61 to 1.33)	(4 RCTs)	Moderate	

^a^A1: risk of bias; ^b^A2:inconsistency; ^c^A3: indirectness; ^d^A4: imprecision; ^e^A5: publication bias; ^f^CI: confidence interval; ^g^RR: risk ratio; ^h^MD: mean difference.

**Patient or population:** Patients who have undergone heart surgery; **Intervention:** High-dose iron; **Comparison:** Placebo.

## Discussion

The present meta-analysis demonstrated that high-dose intravenous iron significantly lowered the risk of blood transfusion following cardiac surgery compared with placebo (RR 0.69) and increased hemoglobin levels after one week following surgery (MD 0.71). However, there were no statistically significant differences in the incidence of adverse events (including death, cardiovascular events, and infections) and the duration of hospitalization between the high-dose iron group and the placebo group.

Recently, four systematic reviews have analyzed the ability of iron supplementation to reduce red blood cell transfusion rates following cardiac surgery. The first review included six non-RCTs and concluded that the existing evidence supporting intravenous iron use prior to cardiac surgery is inadequate [16]. The second meta-analysis included 11studies (seven observational studies and four RCTs) and reported that intravenous iron significantly reduces the number of patients requiring transfusions (RR 0.81); however, the evidence quality was determined to be very low due to substantial risks of bias and inconsistency [17]. The third review examined five RCTs and found no statistically significant difference in postoperative transfusion rates with iron therapy (administered intravenously or orally) (RR 0.86) [15]. The fourth meta-analysis summarized findings from 14 RCTs and reported that intravenous iron lowers the risk of red blood cell transfusion after cardiac surgery (RR 0.77); although subgroup analyses were performed, no distinction was made regarding the dosage of intravenous iron or the combination with adjuvant therapies (e.g., erythropoietin) [14]. In the present meta-analysis, rigorous quality assessment of the included RCTs was performed, and studies involving oral iron and combined adjuvant treatments were excluded. In addition, the present meta-analysis differentiated between doses of intravenous iron. The present findings indicated that high-dose intravenous iron is associated with a reduction in the risk of red blood cell transfusion following cardiac surgery (RR 0.69).

Oral iron supplements are associated with reduced gastrointestinal tolerance and exhibit poor effectiveness in treating iron deficiency in the context of inflammation, which is related to hepcidin lowering iron absorption and preventing iron recycling, thereby limiting erythropoiesis [19]. Robert et al. [31] suggested that intravenous iron supplementation offers better hematological protection than oral iron supplements (control group). The present study further revealed that high-dose intravenous iron supplementation may have potential advantages in reducing the risk of red blood cell transfusion. Donat et al. [32] reported that patients undergoing elective cardiac surgery who receive combination therapy (intravenous iron, subcutaneous erythropoietin alpha, vitamin B12, and oral folate) experience a significant reduction in red blood cell transfusion volume (RR 0.70), which agrees with the present findings regarding the reduction of red blood cell transfusion risk. Compared with the combination therapy regimen, high-dose intravenous iron supplementation provides effective hematological protection, potentially avoiding associated adverse drug reactions, unless patients are diagnosed with renal anemia or have a deficiency of relevant hemoglobin synthesis substrates [33, 34].

According to meta-analysis, Hung et al. [14] reported that intravenous iron supplementation significantly elevates hemoglobin levels on postoperative days 4–10 (MD 0.17) and after three weeks (MD 0.66). This finding disagrees with the present study, primarily due to differing definitions of data extraction principles. In the present study, one week after surgery was established as the critical timepoint for hemoglobin value extraction; the present findings indicated that high-dose intravenous iron supplementation provides a notable advantage in hemoglobin levels one week or more after surgery, including measurements beyond three weeks (MD 0.71). Under normal physiological conditions, hemoglobin synthesis takes approximately one week. A systemic inflammatory response following heart surgery is common and can inhibit hemoglobin synthesis, which delays recovery, thus requiring a longer duration for postoperative hemoglobin normalization [35,36]. However, high-dose intravenous iron supplementation may facilitate this process to some extent. Adequate iron availability is essential for the rapid synthesis of hemoglobin [37]. Although additional validation is needed, these findings suggest that a rapid recovery of hemoglobin after surgery is advantageous for patient prognosis.

In the present study, the funnel plots for two outcome indicators (red cell transfusion rate and postoperative hemoglobin value) exhibited an asymmetric distribution, indicating a certain degree of publication bias, which may lead to overestimation of the therapeutic efficacy of iron supplementation. Subsequent sensitivity analysis demonstrated robust results for these two outcomes, which suggested that such risk was manageable and did not qualitatively alter the assessment of the true efficacy of intravenous iron. However, this objective factor (publication bias) may affect the certainty of evidence, resulting in a downgrade in the overall quality of the evidence [30].

A large systematic review has indicated that intravenous iron supplementation may increase the risk of infection [38]; however, a corresponding increase in this risk was not identified in the present meta-analysis, aligning with the findings of a previous study [39]. Elemental iron is a critical nutrient for bacterial growth, and various bacterial species express iron transport proteins that compete with transferrin, potentially resulting in an increased risk of infection due to iron overload [40]. Although there is insufficient conclusive clinical data, it is advisable to refrain from administering intravenous iron supplementation in cases of acute infection [41].

The present study had several limitations. First, variations in the timing and frequency of intravenous iron administration were not accounted for in the present study, and the present study did not consider the diverse indications for blood transfusion. Garbowski et al. [42] reported that non-transferrin-bound iron, transferrin-bound iron, total serum iron, and hepcidin return to baseline levels within 1–3 days after administration of intravenous iron preparations. With careful consideration of drug safety, administering intravenous iron before and after surgery or continuous administration during the perioperative period may lead to better efficacy, thereby reducing the risk of transfusion. Typically, clinical practice dictates that transfusions are administered to patients with hemoglobin levels below 7–8 g/dL [43]. In the present meta-analysis, the reported transfusion thresholds were 7 g/dL, 8 g/dL, and 9 g/dL in the included studies [22,24,28]. If the transfusion threshold (hemoglobin value) is low, it may reduce the risk of transfusion to some extent, and vice versa. This variability in transfusion standards may have impacted the evaluation of the transfusion risk in the present meta-analysis. Second, some of the studies included in the meta-analysis did not report or lacked data on adverse event rates, which may have impacted the safety evaluation of high-dose intravenous iron in these patients. Third, the present meta-analysis did not address whether alternative blood management strategies were implemented during the perioperative period; these strategies may influence the analysis of transfusion rates and hemoglobin levels. Despite these limitations, the present meta-analysis provides the most recent evidence regarding the efficacy and safety of high-dose intravenous iron for blood conservation in patients undergoing cardiac surgery. These findings can inform clinical decision-making and provide evidence-based support for optimal clinical strategies involving iron preparations.

## Conclusions

High-dose intravenous iron supplementation provides substantial benefits in blood protection for patients undergoing cardiac surgery by reducing the incidence of red blood cell transfusions and enhancing postoperative hemoglobin levels. Although the present meta-analysis indicated that high-dose intravenous iron formulations possess a favorable safety profile, several limitations existed within the study, including the absence of adverse reaction reports in some included studies, missing data, and lower-quality evidence. In addition, the present study did not assess postoperative fatigue and functional recovery among patients. The safety of intravenous iron administration should not be overestimated, particularly in patients with inflammation and chronic diseases, where heightened clinical vigilance regarding potential adverse reactions is essential. In conclusion, when drug safety is carefully considered, the utilization of high-dose intravenous iron for blood protection in cardiac surgery offers significant advantages. However, due to the limitations of recent research and the present meta-analysis, there is an urgent need for large-scale RCTs to investigate the optimal efficacy and safety of high-dose intravenous iron within blood management protocols.

## Supporting information

S1 File All the search strategies for the databases and all the sensitivity analyses of the outcome indicators. (DOCX)

S1 Table PRISMA checklist. (DOCX)

S1 Fig Funnel plot showing red blood cell transfusion rate. (TIF)

S2 Fig Subgroup analysis of red-cell transfusion rate based on preoperative anemia status. (TIF)

S3 Fig Subgroup analysis of the hemoglobin value within one week after surgery based on preoperative anemia status. (TIF)

S4 Fig Subgroup analysis of the hemoglobin value within one week after surgery based on the timing of administration. (TIF)

S5 Fig Funnel plot for hemoglobin levels at postoperative. (TIF)

S6 Fig Subgroup analysis of the length of hospital stay based on the timing of administration and the type of intravenous iron preparation. (TIF)

S7 Fig The funnel plot of mortality rate. (TIF)
