# Supplementary material for: High-dose intravenous iron significantly reduces the risk of red blood cell transfusion and improves postoperative hemoglobin levels after cardiac surgery: A systematic review of randomized controlled trials
Source: PLoS One. 2025 Nov 13;20(11):e0336773. doi: 10.1371/journal.pone.0336773 (PMC12614565; doi:10.1371/journal.pone.0336773)
Supplement: S1 File — (DOCX) [file pone.0336773.s001.docx]

**S1 Table. Search strategies for all databases.**

| The database | Search details |
| --- | --- |
| Pubmed | (("Iron"[MeSH Terms] OR "Iron-56"[Title/Abstract] OR "Iron-56"[Title/Abstract] OR ("ferric carboxymaltose"[Supplementary Concept] OR "iron carboxymaltose"[Title/Abstract] OR "iron dextri maltose"[Title/Abstract] OR "injectafer"[Title/Abstract] OR "Ferinject"[Title/Abstract] OR "VIT-45"[Title/Abstract] OR "VIT-45"[Title/Abstract]) OR ("iron isomaltoside 1000"[Supplementary Concept] OR "iron isomaltoside"[Title/Abstract]) OR ("ferric oxide, saccharated"[MeSH Terms] OR "saccharated ferric oxide"[Title/Abstract] OR ((((("glucaric acid"[MeSH Terms] OR ("glucaric"[All Fields] AND "acid"[All Fields]) OR "glucaric acid"[All Fields] OR "d glucaric acid"[All Fields]) AND ("Iron"[MeSH Terms] OR "Iron"[All Fields])) AND "2"[Title/Abstract]) AND ("sodium chloride"[MeSH Terms] OR ("sodium"[All Fields] AND "chloride"[All Fields]) OR "sodium chloride"[All Fields] OR "salt"[All Fields])) AND "1 1"[All Fields]) OR "Iron-Saccharate"[Title/Abstract] OR "Iron-Saccharate"[Title/Abstract] OR "ferric saccharate"[Title/Abstract] OR (("ferric oxide"[Supplementary Concept] OR "ferric oxide"[All Fields] OR "iron oxide"[All Fields] OR "ferrosoferric oxide"[MeSH Terms] OR ("ferrosoferric"[All Fields] AND "Oxide"[All Fields]) OR "ferrosoferric oxide"[All Fields] OR ("Iron"[All Fields] AND "Oxide"[All Fields])) AND "Saccharated"[Title/Abstract]) OR "iron sucrose"[Title/Abstract] OR "Ferri-Saccharate"[Title/Abstract] OR "Ferri-Saccharate"[Title/Abstract] OR "Hippiron"[Title/Abstract] OR "Venofer"[Title/Abstract] OR ((("Iron"[MeSH Terms] OR "Iron"[All Fields]) AND "III"[All Fields]) AND "hydroxide sucrose complex"[Title/Abstract])) OR ("ferric gluconate"[Supplementary Concept] OR (("ferric gluconate"[Supplementary Concept] OR "ferric gluconate"[All Fields]) AND "trihydrate"[Title/Abstract]) OR "ferrlecit 100"[Title/Abstract] OR "Ferrlecit"[Title/Abstract] OR (((("sodium"[MeSH Terms] OR "sodium"[All Fields] OR "sodiums"[All Fields]) AND ("Iron"[MeSH Terms] OR "Iron"[All Fields])) AND "III"[All Fields]) AND "gluconate"[Title/Abstract]) OR (("ferric gluconate"[Supplementary Concept] OR "ferric gluconate"[All Fields]) AND "sodium salt"[Title/Abstract]) OR "sodium ferrigluconate"[Title/Abstract] OR (("ferric gluconate"[Supplementary Concept] OR "ferric gluconate"[All Fields]) AND "anhydrous"[Title/Abstract])) OR ("Iron-Dextran Complex"[MeSH Terms] OR "Iron-Dextran Complex"[Title/Abstract] OR "Ferridextran"[Title/Abstract] OR "dextran iron complex"[Title/Abstract] OR "dextran iron complex"[Title/Abstract] OR "Imposil"[Title/Abstract] OR "Imferon"[Title/Abstract] OR "Dextrofer"[Title/Abstract] OR "Dexferrum"[Title/Abstract] OR "Feosol"[Title/Abstract] OR "Icar"[Title/Abstract] OR "InFed"[Title/Abstract])) AND ("Thoracic Surgery"[MeSH Terms] OR "surgery thoracic"[Title/Abstract] OR "surgery cardiac"[Title/Abstract] OR "cardiac surgery"[Title/Abstract] OR "surgery heart"[Title/Abstract] OR "heart surgery"[Title/Abstract] OR ("Coronary Artery Bypass"[MeSH Terms] OR "artery bypass coronary"[Title/Abstract] OR ((("arterialization"[All Fields] OR "arterializations"[All Fields] OR "arterialize"[All Fields] OR "arterialized"[All Fields] OR "arterializing"[All Fields] OR "arterially"[All Fields] OR "arterials"[All Fields] OR "arterie"[All Fields] OR "arteries"[MeSH Terms] OR "arteries"[All Fields] OR "arterial"[All Fields] OR "arteris"[All Fields] OR "Artery"[All Fields] OR "arterious"[All Fields] OR "artery s"[All Fields] OR "arterys"[All Fields]) AND ("Bypass"[All Fields] OR "bypassed"[All Fields] OR "Bypasses"[All Fields] OR "bypassing"[All Fields])) AND "Coronary"[Title/Abstract]) OR (("Bypass"[All Fields] OR "bypassed"[All Fields] OR "Bypasses"[All Fields] OR "bypassing"[All Fields]) AND "coronary artery"[Title/Abstract]) OR "coronary artery bypasses"[Title/Abstract] OR "coronary artery bypass grafting"[Title/Abstract] OR "coronary artery bypass surgery"[Title/Abstract] OR "aortocoronary bypass"[Title/Abstract] OR "aortocoronary bypasses"[Title/Abstract] OR "bypass aortocoronary"[Title/Abstract] OR (("Bypass"[All Fields] OR "bypassed"[All Fields] OR "Bypasses"[All Fields] OR "bypassing"[All Fields]) AND "Aortocoronary"[Title/Abstract]) OR "bypass surgery coronary artery"[Title/Abstract] OR "bypass coronary artery"[Title/Abstract]) OR ("Heart Valve Prosthesis Implantation"[MeSH Terms] OR "implantation heart valve prosthesis"[Title/Abstract]))) AND (randomizedcontrolledtrial[Filter]) |
| Cochrane | #1 MeSH descriptor: [Iron] explode all trees 3218  #2 MeSH descriptor: [Ferric Oxide, Saccharated] explode all trees 238  #3 MeSH descriptor: [Iron-Dextran Complex] explode all trees 78  #4 (ferric carboxymaltose OR iron carboxymaltose OR iron dextri-maltose OR injectafer OR Ferinject OR VIT-45 OR VIT 45 OR iron isomaltoside 1000 OR iron isomaltoside OR ferric gluconate OR ferric gluconate trihydrate OR Ferrlecit 100 OR Ferrlecit OR sodium iron(III)gluconate OR ferric gluconate, sodium salt OR sodium ferrigluconate OR ferric gluconate anhydrous OR Iron-56 OR Iron 56 OR Saccharated Ferric Oxide OR Iron-Saccharate OR Iron Saccharate OR Ferric Saccharate OR Iron Oxide (Saccharated) OR Iron Sucrose OR Ferri-Saccharate OR Ferri Saccharate OR Hippiron OR Venofer OR Iron Dextran Complex OR Ferridextran OR Dextran-Iron Complex OR Dextran Iron Complex OR Imposil OR Imperon OR Imferon OR Dextrofer OR Dexferrum OR Feosol OR Hematran OR Icar OR Imfergen OR InFed OR Norferan):ti,ab,kw (Word variations have been searched) 5089  #5 #1 OR #2 OR #3 OR #4 7806  #6 MeSH descriptor: [Thoracic Surgery] explode all trees 238  #7 MeSH descriptor: [Coronary Artery Bypass] explode all trees 6710  #8 MeSH descriptor: [Heart Valve Prosthesis Implantation] explode all trees 1447  #9 (Surgery, Thoracic OR Surgery, Cardiac OR Cardiac Surgery OR Surgery, Heart OR Heart Surgery OR Artery Bypass, Coronary OR Artery Bypasses, Coronary OR Bypasses, Coronary Artery OR Coronary Artery Bypasses OR Coronary Artery Bypass Grafting OR Coronary Artery Bypass Surgery OR Aortocoronary Bypass OR Aortocoronary Bypasses OR Bypass, Aortocoronary OR Bypasses, Aortocoronary OR Bypass Surgery, Coronary Artery OR Bypass, Coronary Artery OR Implantation, Heart Valve Prosthesis):ti,ab,kw (Word variations have been searched) 62161  #10 #6 OR #7 OR #8 OR #9 62277  #11 #5 AND #10 146 |
| Embase | Query('iron'/exp OR 'iron' OR 'iron saccharate'/exp OR 'iron saccharate' OR 'iron dextran'/exp OR 'iron dextran' OR 'ferric carboxymaltose':ab,kw,ti OR 'iron carboxymaltose':ab,kw,ti OR 'iron dextri-maltose':ab,kw,ti OR 'injectafer':ab,kw,ti OR 'ferinject':ab,kw,ti OR 'vit-45':ab,kw,ti OR 'vit 45':ab,kw,ti OR 'iron isomaltoside 1000':ab,kw,ti OR 'iron isomaltoside':ab,kw,ti OR 'ferric gluconate':ab,kw,ti OR 'ferric gluconate trihydrate':ab,kw,ti OR 'ferrlecit 100':ab,kw,ti OR 'ferrlecit':ab,kw,ti OR 'sodium iron(iii)gluconate':ab,kw,ti OR 'ferric gluconate, sodium salt':ab,kw,ti OR 'sodium ferrigluconate':ab,kw,ti OR 'ferric gluconate anhydrous':ab,kw,ti OR 'iron-56':ab,kw,ti OR 'iron 56':ab,kw,ti OR 'saccharated ferric oxide':ab,kw,ti OR 'iron-saccharate':ab,kw,ti OR 'iron saccharate':ab,kw,ti OR 'ferric saccharate':ab,kw,ti OR 'iron oxide (saccharated)':ab,kw,ti OR 'iron sucrose':ab,kw,ti OR 'ferri-saccharate':ab,kw,ti OR 'ferri saccharate':ab,kw,ti OR 'hippiron':ab,kw,ti OR 'venofer':ab,kw,ti OR 'iron dextran complex':ab,kw,ti OR 'ferridextran':ab,kw,ti OR 'dextran-iron complex':ab,kw,ti OR 'dextran iron complex':ab,kw,ti OR 'imposil':ab,kw,ti OR 'imperon':ab,kw,ti OR 'imferon':ab,kw,ti OR 'dextrofer':ab,kw,ti OR 'dexferrum':ab,kw,ti OR 'feosol':ab,kw,ti OR 'hematran':ab,kw,ti OR 'icar':ab,kw,ti OR 'imfergen':ab,kw,ti OR 'infed':ab,kw,ti OR 'norferan':ab,kw,ti) AND ('heart valve prosthesis implantation' OR 'heart valve replacement' OR 'coronary artery bypass graft'/exp OR 'coronary artery bypass graft' OR 'thorax surgery'/exp OR 'thoracic surgery' OR 'surgery, thoracic':ab,kw,ti OR 'surgery, cardiac':ab,kw,ti OR 'cardiac surgery':ab,kw,ti OR 'surgery, heart':ab,kw,ti OR 'heart surgery':ab,kw,ti OR 'artery bypass, coronary':ab,kw,ti OR 'artery bypasses, coronary':ab,kw,ti OR 'bypasses, coronary artery':ab,kw,ti OR 'coronary artery bypasses':ab,kw,ti OR 'coronary artery bypass grafting':ab,kw,ti OR 'coronary artery bypass surgery':ab,kw,ti OR 'aortocoronary bypass':ab,kw,ti OR 'aortocoronary bypasses':ab,kw,ti OR 'bypass, aortocoronary':ab,kw,ti OR 'bypasses, aortocoronary':ab,kw,ti OR 'bypass surgery, coronary artery':ab,kw,ti OR 'bypass, coronary artery':ab,kw,ti OR 'implantation, heart valve prosthesis':ab,kw,ti) AND 'randomized controlled trial' |

| **S2 Table. Sensitivity analysis of red blood cell transfusion.** | | | | |
| --- | --- | --- | --- | --- |
| Excluded studies | The combined value of the remaining studies | | | |
|  | P | I² | RR | 95% CI |
| 2015 Johansson | 0.01 | 67% | 0.68 | 0.51, 0.92 |
| 2019 Xu | 0.02 | 66% | 0.69 | 0.51, 0.94 |
| 2022 Shokri | 0.01 | 16% | 0.81 | 0.68, 0.95 |
| 2022 Song | 0.006 | 57% | 0.61 | 0.42, 0.87 |
| 2023 Friedman | 0.03 | 58% | 0.72 | 0.53, 0.97 |
| 2023 Houry | 0.02 | 66% | 0.61 | 0.41, 0.92 |
| 2023 Kim | 0.01 | 67% | 0.68 | 0.50, 0.93 |

| **S3 Table. Sensitivity analysis of hemoglobin values within one week after surgery.** | | | | |
| --- | --- | --- | --- | --- |
| Excluded studies | The combined value of the remaining studies | | | |
|  | P | I² | MD | 95% CI |
| 2023 Kim | 0.19 | 95 | 0.53 | -0.25, 1.31 |
| 2023 Houry | 0.25 | 95 | 0.51 | -0.36, 1.38 |
| 2023 Friedman | 0.14 | 94 | 0.59 | -0.18, 1.36 |
| 2022 Shokri | 0.09 | 0 | 0.18 | -0.02, 0.38 |
| 2019 Xu | 0.21 | 95 | 0.53 | -0.31, 1.35 |

| **S4 Table. Sensitivity analysis of hemoglobin values at one week or later after surgery.** | | | | |
| --- | --- | --- | --- | --- |
| Excluded studies | The combined value of the remaining studies | | | |
|  | P | I² | MD | 95% CI |
| 2023 Kim | 0.00001 | 52 | 0.62 | 0.36, 0.88 |
| 2023 Houry | 0.0002 | 69 | 0.70 | 0.33, 1.08 |
| 2022 Song | 0.0001 | 67 | 0.68 | 0.33, 1.03 |
| 2023 Friedman | 0.0001 | 71 | 0.73 | 0.36, 1.10 |
| 2022 Shokri | 0.00001 | 24 | 0.81 | 0.58, 1.04 |
| 2019 Xu | 0.0001 | 70 | 0.75 | 0.40, 1.11 |

| **S5 Table. Sensitivity analysis of hospitalization duration.** | | | | |
| --- | --- | --- | --- | --- |
| Excluded studies | The combined value of the remaining studies | | | |
|  | P | I² | MD | 95% CI |
| 2019 Xu | 0.3 | 73 | -2.5 | -7.27, 2.27 |
| 2022 Shokri | 0.96 | 0 | 0.02 | -0.83, 0.87 |
| 2023 Kim | 0.31 | 99 | -2.19 | -6.45, 2.07 |

| **S6 Table. Sensitivity analysis of mortality rate.** | | | | |
| --- | --- | --- | --- | --- |
| Excluded studies | The combined value of the remaining studies | | | |
|  | P | I² | RR | 95% CI |
| 2015 Johansson | 0.41 | 0 | 0.66 | 0.25, 1.77 |
| 2019 Xu | 0.61 | 0 | 0.76 | 0.27, 2.16 |
| 2022 Shokri | 0.49 | 0 | 0.66 | 0.20, 2.18 |
| 2022 Song | 0.25 | 0 | 0.53 | 0.18, 1.56 |
| 2023 Friedman | 0.52 | 0 | 0.71 | 0.25, 2.01 |
| 2023 Houry | 0.37 | 0 | 0.62 | 0.22, 1.78 |
| 2023 Kim | 0.52 | 0 | 0.70 | 0.24, 2.07 |

| **S7 Table. Sensitivity analysis of infection rate.** | | | | |
| --- | --- | --- | --- | --- |
| Excluded studies | The combined value of the remaining studies | | | |
|  | P | I² | RR | 95% CI |
| 2015 Johansson | 0.94 | 0 | 0.97 | 0.47, 2.00 |
| 2022 Shokri | 0.23 | 0 | 1.53 | 0.76, 3.09 |
| 2022 Song | 0.6 | 24 | 1.23 | 0.57, 2.68 |
| 2023 Friedman | 0.5 | 22 | 1.37 | 0.54, 3.46 |

| **S8 Table. Sensitivity analysis of the incidence rate of cardiovascular events.** | | | | |
| --- | --- | --- | --- | --- |
| Excluded studies | The combined value of the remaining studies | | | |
|  | P | I² | RR | 95% CI |
| 2015 Johansson | 0.32 | 0 | 0.78 | 0.49, 1.26 |
| 2022 Shokri | 0.85 | 0 | 0.96 | 0.63, 1.46 |
| 2022 Song | 0.61 | 0 | 0.90 | 0.61, 1.33 |
| 2023 Friedman | 0.97 | 0 | 0.99 | 0.57, 1.71 |
